# Subsequent shunt closure after targeted medical therapy can be an effective strategy for secundum atrial septal defect with severe pulmonary arterial hypertension: two case reports: Strategy for ASD with Severe PAH

**DOI:** 10.1007/s00380-013-0351-0

**Published:** 2013-04-18

**Authors:** Yu Taniguchi, Noriaki Emoto, Kazuya Miyagawa, Kazuhiko Nakayama, Hiroto Kinutani, Hidekazu Tanaka, Toshiro Shinke, Kenji Okada, Yutaka Okita, Ken-ich Hirata

**Affiliations:** 1 Division of Cardiovascular Medicine, Department of Internal Medicine, Kobe University Graduate School of Medicine, 7-5-1 Kusunoki Chuo, 650-0017 Kobe, Japan; 2 Division of Cardiovascular Surgery, Department of Surgery, Kobe University Graduate School of Medicine, Kobe, Japan

**Keywords:** Atrial septal defect, Pulmonary arterial hypertension, Pulmonary vasodilators, Shunt closure

## Abstract

Secundum atrial septal defect (ASD) is the most common form of congenital heart disease in adults. Surgical and transcatheter closures of ASD are widely accepted therapeutic approaches. In patients with severe pulmonary arterial hypertension (PAH), however, the closure of the defect is still controversial. We report two cases of ASD patients with severe PAH successfully repaired subsequent to effective medical therapy. Subsequent shunt closure after targeted medical therapy can be an effective strategy in selected ASD patients with severe PAH.

## Introduction

Secundum atrial septal defect (ASD) is one of the most common forms of congenital heart disease (CHD) in adults. With advancing years, patients become symptomatic and may develop pulmonary arterial hypertension (PAH). Surgical and transcatheter closures of ASD are widely accepted treatments. In patients with severe PAH, however, closure presents the risk of provoking right ventricular failure and pulmonary hypertensive crisis [1]. Targeted medical therapies have been established for the treatment of PAH associated with CHD, and they can potentially modify the indication of ASD closure in patients with severe PAH [1]. We report two cases of patients with secundum ASD and severe PAH successfully treated with surgical and transcatheter closures subsequent to effective combination medical therapy.

## Case 1

**Fig. 1:**
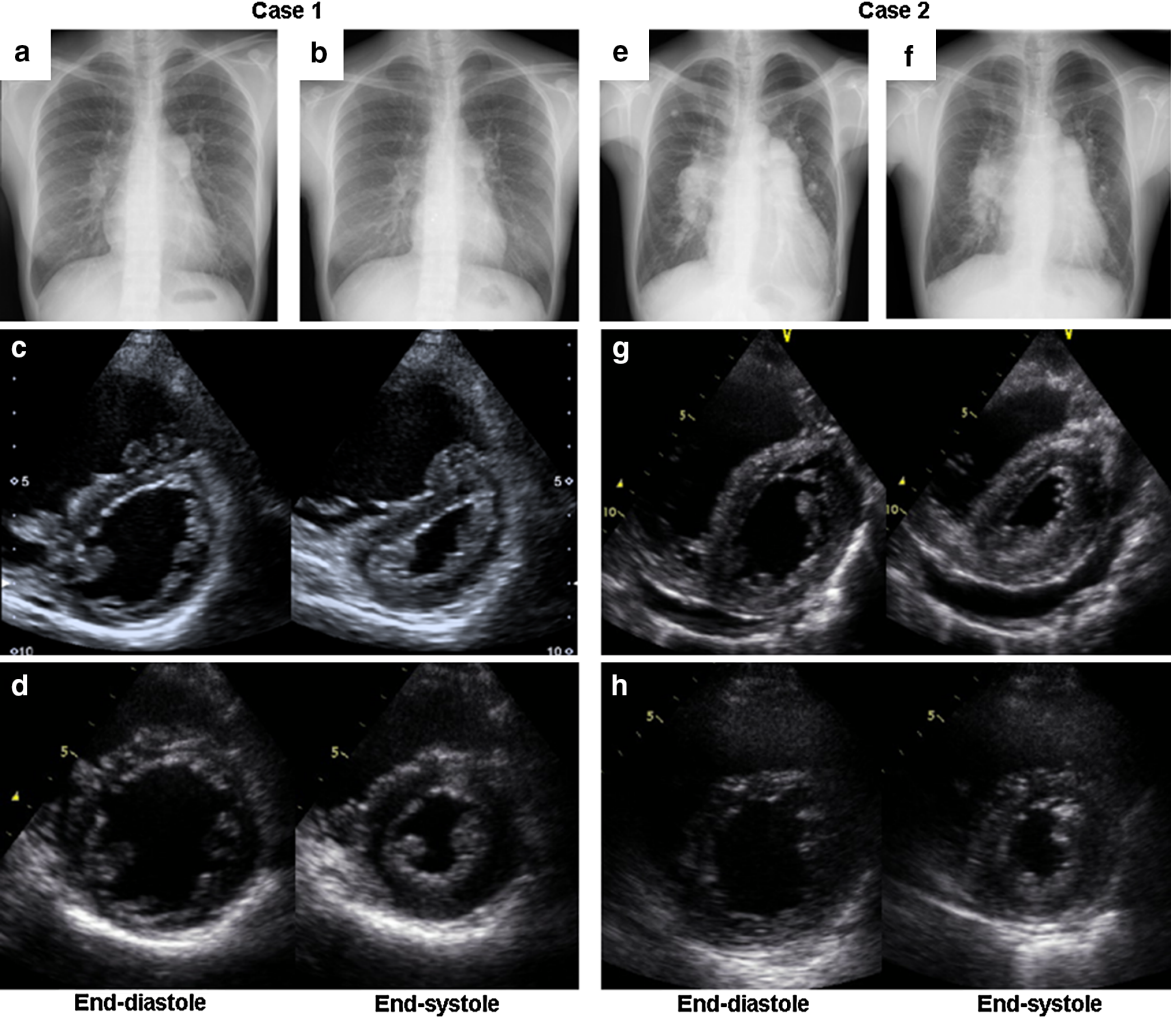
Chest X-ray and trans-thoracic echocardiogram. Case 1 (**a**–**d**) and case 2 (**e**–**h**). Cardiomegaly and ventricular overload were dramatically improved after closure (**b**, **d**, **f**, **h**) compared with those on admission (**a**, **c**, **e**, **g**)

A 31-year-old woman was referred to our hospital because of exertional dyspnea and syncope. She had felt exertional dyspnea about 2 years ago. The World Health Organization (WHO) functional class was III on admission. The 6-min walk distance was 20 m terminated by syncope. She had no familial history of PAH. Echocardiography revealed right ventricular dilatation and hypertrophy with secundum ASD (15 mm in diameter) with left-to-right shunt. Right heart catheterization (RHC) showed elevated pulmonary artery pressure (PAP) and pulmonary vascular resistance (PVR) of 87/30 mmHg (mean 57 mmHg) and 697 dyn s/cm^5^, respectively. Cardiac output was 5.71 l/min estimated by the Fick method. The Qp/Qs ratio was 1.32. We started supplemental oxygen therapy and medical therapy with bosentan (250 mg/day) and sildenafil (60 mg/day). Two months later, RHC revealed the improvement of PAP and PVR, 60/21 mmHg (mean 35 mmHg) and 291 dyn s/cm^5^. Although medical therapy was effective, the Qp/Qs ratio increased to 2.19 and moderate dyspnea remained. We thought that continuation of medical therapy might further reduce PAP and PVR, but subsequently increase shunt flow, which would insult the pulmonary circulation. Therefore, we performed transcatheter closure 5 months after the initiation of medical therapy. After repair, symptoms had improved to WHO functional class I without oxygen administration, and the chest X-ray and echocardiography demonstrated the improvement of right ventricular overload (Fig. 1). The combination medical therapy was continued after the procedure. RHC performed 6 months after closure revealed that PAP was 29/15 mmHg (mean 22 mmHg) and PVR 197 dyn s/cm^5^.

## Case 2

A 47-year-old man was admitted to our hospital because of leg edema and exertional dyspnea. He was diagnosed with ASD in childhood, but felt no symptom until 3 years ago. WHO functional class was III on admission. Echocardiography revealed right ventricular dilatation, hypertrophy and pericardial effusion with large secundum ASD (36 mm in diameter) with left-to-right shunt. RHC showed PAP of 93/29 mmHg (mean 60 mmHg), PVR of 539 dyn s/cm^5^ and Qp/Qs ratio of 1.51. We started supplemental oxygen therapy and medical therapy with bosentan (250 mg/day), tadarafil (40 mg/day) and beraprost (60 μg/day). The symptoms improved within 1 month to WHO functional class II. PAP and PVR decreased to 58/28 mmHg (mean 39 mmHg) and 109 dyn s/cm^5^, respectively, while the Qp/Qs ratio consequently increased to 3.55. We therefore performed surgical closure 2 months after starting the medical therapy. We continued medical therapy with bosentan (250 mg/day), tadarafil (40 mg/day) and beraprost (60 μg/day) after closure. The symptoms had improved to WHO functional class I without oxygen administration. The chest X-ray and echocardiography showed the improvement of the right ventricular overload (Fig. 1). RHC revealed a PAP of 29/21 mmHg (mean 24 mmHg), PVR of 190 dyn s/cm^5^ and Qp/Qs ratio of 1.07.

## Discussion

Surgical and transcatheter closures are therapeutic standards for ASD. There are few studies reporting the efficacy and safety of the closure in patients with severe PAH [2], and the benefit of closure remains controversial. Steele et al. [3] have shown that high PVR is associated with poor prognosis in surgically treated patients. Hindi et al. [4] have suggested that severe PAH manifested as Qp/Qs <0.7 and PVR > 7 Woods units are contraindications for transcatheter closure. In these situations, pulmonary vascular disease is thought to be irreversible, and closure of the defect might induce right ventricular failure and pulmonary hypertensive crisis [1].

In PAH associated with CHD, targeted medical therapy has been standardized and may induce reverse remodeling of pulmonary arteriopathy [5]. Based on these characteristics, medical therapy may have the potential to modify the operability of ASD. The so-called ‘treat-and-repair’ approach, which implements medical therapy ahead of surgical repair, has been discussed [1].

Because of the severity of symptoms, particularly the high PAP and PVR, we first considered our patients inoperable and gave priority to medical therapy. With initial combination therapy, we successfully decreased PAP and PVR in both patients. This effect was achieved in the short term, but the symptoms remained. Further therapy was needed. Given the increased Qp/Qs ratio, additional vasodilators could have induced a further increase in left-to-right shunt and PAP, which would have led to progression of pulmonary arteriopathy. Since the reduction of PVR was rapid, we assumed a strong vasoreactivity of pulmonary arteries. We thus considered the patients operable and decided to close the defects to further reduce PAP. Finally, normal hemodynamics and asymptomatic conditions were attained.

Several case reports also mentioned the efficacy of treat-and-repair approaches [6, 7]. In contrast to these previous studies, we undertook repair after a short-term effective combination medical therapy (2–5 months). If pulmonary vascular reactivity is preserved, medical therapy will decrease PVR, which may increase pulmonary flow through the defect and insult the pulmonary vasculature [1]. Therefore, in case of preserved pulmonary vasoreactivity, early closure of the defect may be safe and prevent further pulmonary vascular remodeling.

Although Eisenmenger syndrome shares pathological manifestations with idiopathic PAH (IPAH), the prognosis is known to be better than that of IPAH [8]. As a reason for this, a right-to-left shunt might act as a relief valve for the right ventricle [1]. Similarly, IPAH patients with an incidental ASD also have a better prognosis. These observations argue against the operability of ASD patients with severe PAH because the closure may worsen the prognosis. In case 1, the small size of the defect may indicate that the patient suffered from IPAH with an incidental ASD. Nevertheless, the ASD could be closed successfully, and normal PAP levels were achieved, which may have hindered the progression of pulmonary vascular disease. Based on our cases and previous reports [6, 7, 9], consistent left-to-right shunt and preserved vasoreactivity should be considered prerequisites for safe and effective repair of ASD in PAH patients.

We presented two cases of successful treatment for ASD and severe PAH with short-term powerful combination medical therapy ahead of the closure. Even though this approach cannot be generalized, subsequent shunt closure after medical therapy should be considered effective in selected patients with ASD and severe PAH. Clinical studies are required to evaluate the efficacy and safety of this strategy.
